# Mesenchymal Stromal Cells Respond to SARS-CoV-2 Peptides and Exhibit Altered T-Cell Regulatory Capacity

**DOI:** 10.3390/cells15070592

**Published:** 2026-03-26

**Authors:** Sabrina Summer, Hermann Maximilian Wolf, Viktoria Weber, Michael B. Fischer

**Affiliations:** 1 Center for Experimental Medicine, Department for Biomedical Research, University for Continuing Education Krems, 3500 Krems, Austria; michael.fischer@donau-uni.ac.at; 2 Faculty of Medicine, Sigmund Freud Private University, 1020 Vienna, Austria; hermann.wolf@med.sfu.ac.at; 3 Center for Biomedical Technology, Department for Biomedical Research, University for Continuing Education Krems, 3500 Krems, Austria; viktoria.weber@donau-uni.ac.at; 4 Department for Blood Group Serology and Transfusion Medicine, Medical University of Vienna, 1090 Vienna, Austria

**Keywords:** mesenchymal stromal cells, T-cells, SARS-CoV-2 peptides, COVID-19, immunoregulation

## Abstract

**Highlights:**

**What are the main findings?**

- Severe acute respiratory syndrome coronavirus (SARS-CoV)-2-related stimuli alter mesenchymal stromal cell (MSC)-mediated immunoregulation of T-cells through the activation of the Toll-like receptor (TLR)4 pathway.
- Combined exposure of MSCs to SARS-CoV-2 peptides and lipopolysaccharide (LPS) indicates potential reciprocal signaling interactions.

**What are the implications of the main findings?**

- SARS-CoV-2-associated inflammatory signals can compromise the immunosuppressive capacity of MSCs.
- TLR4 emerges as a mechanistic pathway of MSCs in SARS-CoV-2 peptide recognition.

**Abstract:**

Background: MSCs possess strong immunoregulatory properties and play a central role in maintaining immune homeostasis by limiting inflammatory responses. Their function is highly plastic and influenced by environmental cues, including viral signals. How SARS-CoV-2-derived antigens affect MSC immunoregulation remains incompletely understood. This study aimed to investigate the impact of SARS-CoV-2 peptides on MSC-mediated immune modulation of T-cells. Methods: MSCs were stimulated directly with SARS-CoV-2 spike protein S peptides or cocultured with SARS-CoV-2 peptide-activated T-cells. TLR4 surface expression and receptor downstream signaling were assessed to evaluate pathway activation. MSC immunoregulatory function was analyzed by measuring suppression of TNF-α and IFN-γ expression and induction of CD4^+^FOXP3^+^ regulatory T-cells. TLR4 inhibition and lipopolysaccharide (LPS) stimulation were used to examine pathway specificity and interaction. Results: SARS-CoV-2 peptides activated TLR4-associated signaling in MSCs, increasing TLR4 expression and NF-κB phosphorylation. Peptide-treated MSCs showed impaired suppression of pro-inflammatory cytokines and reduced induction of regulatory T-cells. TLR4 inhibition prevented these effects. LPS induced similar effects, while combining LPS and peptide stimulation partially restored physiological T-cell cytokine suppression. Conclusions: SARS-CoV-2 peptides modulate MSC immunoregulatory function on T-cells via TLR4-dependent mechanisms.

## 1. Introduction

The immunomodulatory role of mesenchymal stromal cells (MSCs) has gained increasing recognition, with these cells acting as both sensors and effectors within inflammatory microenvironments [1,2].

As immune regulators, MSCs exhibit a dual function. They can either promote immune activation in response to injury or infection [3] or exert potent immunosuppressive effects to prevent excessive immune responses and maintain homeostasis [4]. This modulatory behavior is highly dependent on the surrounding tissue niche and environmental factors and may occur via direct cell-to-cell interactions or the secretion of soluble mediators, such as cytokines, chemokines and growth factors [5]. Central to their anti-inflammatory properties are soluble factors, such as prostaglandin E2 (PGE2), transforming growth factor-β (TGF-β), and indolamine-2,3-dioxygenase (IDO), as well as their interactions with immune cell subsets, particularly the induction and expansion of regulatory T-cells (Tregs) expressing forkhead box P3 (FOXP3) [6,7,8,9,10]. In particular, IDO is considered a key molecule mediating the balance between the immunostimulatory and immunosuppressive functions of MSCs [11]. Toll-like receptor (TLR)-3 activation can further enhance their anti-inflammatory activity by upregulating Delta-like 1-mediated Notch signaling [12,13].

Primarily, MSCs differentiate along the mesodermal lineages into osteocytes, chondrocytes, adipocytes, and fibroblasts and are therefore found in a range of tissues such as placental tissues, bone, cartilage, and adipose tissue. There is evidence that under specific conditions, MSCs exhibit plasticity allowing for limited differentiation into ectodermal and endodermal lineages as well [14,15]. These lineage commitment processes are governed by distinct transcription factors and signaling cascades. While differentiation into mesodermal lineages such as osteogenic, chondrogenic, and adipogenic fates is well established, reported trans-differentiation into non-mesodermal cell types remains controversial, as most evidence comes from in vitro models, and its relevance in vivo is still uncertain [14]. Further, MSCs of different tissue origin display distinct gene expression patterns, which may contribute to their variable differentiation capacities. This source-dependent functional heterogeneity is highly relevant to their biological role, as MSCs are known to play pivotal roles in tissue repair [16], angiogenesis [17,18] and immune regulation [19,20].

It is generally assumed that MSCs exert low immunogenicity primarily due to their absence of major histocompatibility complex (MHC) class II molecules. However, emerging evidence suggests that they can upregulate MHC class II under specific inflammatory conditions. Interferon (IFN)-γ stimulation can induce upregulation of MHC class II, particularly HLA-DR, as well as costimulatory molecules such as the intercellular adhesion molecule (ICAM)-1 and vascular cellular adhesion molecule (VCAM)-1 [21,22]. This enables MSCs to interact directly with CD4^+^ T-cells, potentially presenting antigens and influencing the adaptive immune response. Their antigen-presenting capacity, however, appears to be incomplete or regulatory in nature, as MSCs express lower levels of key co-stimulatory factors such as CD80 than professional antigen-presenting cells (APCs), thereby favoring T-cell anergy or tolerance rather than activation [23,24].

Given their combination of regenerative and immunomodulatory capacity, MSCs have potential in mitigating hyper-inflammation and supporting tissue repair in coronavirus disease 2019 (COVID-19) patients [25,26,27].

The COVID-19 pandemic has underscored the importance of understanding the immunopathology of viral infections. Severe acute respiratory syndrome coronavirus 2 (SARS-CoV-2) triggers a host immune response through pattern recognition receptors (PRRs) including surface receptors such as TLRs, and cytosolic pattern recognition receptors such as retinoic acid-inducible gene (RIG)-I-like receptors, the melanoma differentiation-associated protein (MDA)-5 and C-type lectin receptors (CLRs) [28,29,30]. In severe cases, this results in a dysregulated immune response and extensive release of cytokines and other pro-inflammatory factors, contributing to tissue damage and multiorgan failure [31,32]. Compared to other TLR family members such as TLR2, TLR4 has been proposed to play an important role in the recognition of SARS-CoV-2 components and the amplification of inflammatory signaling pathways associated with severe COVID-19 [33,34]. In addition, endosomal TLR3, TLR7, and TLR8 contribute to antiviral immune responses by detecting viral nucleic acids and their derivatives [35,36,37]. Among these receptors, TLR3 has been particularly associated with the inflammatory outcomes observed in patients [38,39]. Although this receptor is primarily expressed intracellularly, evidence suggests that TLR3 can, under certain conditions, translocate to the cell surface [40]. This relocation allows the receptor to detect extracellular RNA released from neighboring damaged cells, thereby further perpetuating inflammation [36]. An important but so far underexplored question is whether MSCs can directly sense SARS-CoV-2 viral components and how this recognition might alter their immunomodulatory behavior. MSCs express TLRs on their surface that can potentially recognize these viral components: among others, TLR-4, a receptor mostly implicated in lipopolysaccharide (LPS) recognition and inflammatory responses [27]. TLR4-mediated signaling in MSCs has been linked to the induction of a pro-inflammatory phenotype [13], potentially influencing the therapeutic role of MSCs in severe COVID-19 infections [34,41]. MSCs express also other viral sensing receptors such as RIG-1 and MDA-5, which could detect viral RNA within the cell. This study, however, focuses on the ability of MSCs to recognize viral peptides on their surface.

Here, we investigated the capacity of MSCs to recognize SARS-CoV-2-derived peptides [42,43] and to assess how these interactions influence their immunomodulatory function on T-cells. Antigen-specific T-cells are central to the adaptive immune response in COVID-19, where they coordinate pathogen-specific immunity but can also contribute to excessive inflammation. MSCs have been extensively studied for their reciprocal interactions with T-cells under inflammatory conditions [44,45,46] and are known to regulate immune responses by promoting the differentiation and stability of Tregs. Tregs play a critical role in controlling immune activation and limiting inflammation-mediated tissue damaging. Dysregulated Treg responses have been associated with disease severity in COVID-19 [47]. By supporting Treg induction and function, MSCs may contribute to the resolution of excessive immune responses during viral infection. Understanding how SARS-CoV-2-associated signals affect MSC-T-cell crosstalk is therefore essential for clarifying the role of MSCs in shaping immune regulation during COVID-19 and related inflammatory conditions.

## 2. Materials and Methods

### 2.1. Human Donor Samples

The study was conducted in accordance with the Declaration of Helsinki and approved by the ethics committee of the University for Continuous Education Krems, ethics vote number EK GZ 13/2015-2025, and the Ethic Commission of Lower Austria, ethics vote number GSl-EK-4/3122015. Written informed consent was acquired from all donors. The study included 16 blood donators and 18 placental tissue donators (Table 1).

### 2.2. Isolation of T-Cells from Human Whole Blood

Human whole blood was freshly drawn from healthy donors at the University for Continuous Education Krems (EK GZ 13/2015-2025) into vacutainer tubes (Vacuette, Greiner Bio-One, Kremsmünster, Austria) containing sodium citrate following written informed consent. Leucocyte reduction chambers, obtained as medical waste during single platelet apheresis of healthy donors, were provided by the Department of Transfusion Medicine and Blood Group Serology at the AKH Vienna. Mononuclear cells (MNCs) were isolated using gradient density centrifugation (Lymphoprep, Stemcell Technologies, Köln, Germany). MNCs were washed with phosphate-buffered saline (PBS, Gibco, ThermoFisher, Waltham, MA, USA) and used for the isolation of naïve T-cells using the human T-cell isolation kit (Stemcell Technologies), cultured in RPMI-Medium 1640 medium (Gibco) containing 2 mM L-glutamine, 100 U/mL penicillin and 100 mg/mL streptomycin (Invitrogen, ThermoFisher, Carlsbad, CA, USA) and supplemented with 10% heat inactivated fetal calf serum (FBS, Gibco, ThermoFisher) at 37 °C in humidified atmosphere containing 5% CO_2_ at a concentration of 1 × 10^6^ cells/mL. The efficiency of T-cell isolation was tested by measuring CD3^+^ cell enrichment by flow cytometry. All experiments were performed with freshly isolated T-cells from preparations with an isolation efficiency above 90%. When indicated, T-cells were pre-incubated over night with staphylococcal enterotoxin B (SEB) at a final concentration of 1 μg/mL [48], ImmunoCult Human CD3/CD28 T Cell Activator (Stemcell Technologies), using 25 µL/mL medium and PepTivator SARS-CoV-2 Prot_S (Miltenyi, Bergisch-Gladbach, Germany) [42] at a concentration of 1 μg of peptides/mL. Controls were incubated in culture medium only. The PepTivator SARS-CoV-2 Prot_S pool contains 15-mer peptides with a 11 aa overlap covering the immunodominant sequence domains aa 304–338, 421–475, 492–519, 683–707, 741–770, 785–802, and 885–1273 of the surface (spike) glycoprotein of SARS-CoV-2. The sequences are listed in Table S1.

### 2.3. Isolation of Amniotic Mesenchymal Stem Cells from Human Placental Tissue

MSCs were isolated from human placental tissue within the first 24 h after birth and cultured in MSC growth medium (MSCGM, Lonza, Basel, Switzerland) as described previously [49]. Each MSC preparation was routinely analyzed in passage 1 for the expression of the surface markers CD73, CD90 and CD105, as well as CD31, CD34, CD44 and CD45 (Table S2, Figure S1) by flow cytometry using the CytoFlex LX (Beckman Coulter GmbH, Brea, CA, USA) in accordance with the ISCT guidelines [50], as previously described [18]. Placental tissues were obtained from healthy delivering women in accordance with the Austrian Hospital Act (KAG 1982) after written informed consent. The study was approved by the Ethic Commission of Lower Austria (GSl-EK-4/3122015).

### 2.4. Coculture Assays

MSCs were seeded 24 h before treatment or co-culturing in 6-well plates and T-25 flasks, respectively. For coculture assays, T-cells and MSCs were seeded at an effector-to-target (E:T) ratio of 1:20. After stimulation, T-cells were added to the MSCs in MSCBM medium supplemented with 10% FBS. After 24 h at 37 °C and 5% CO_2_, cells were collected, washed once with PBS, and used for further analysis. For intracellular flow cytometry, cells were treated with Brefeldin A (Biolegend, San Diego, CA, USA) for 4 h before harvesting. MSCs were stimulated at a concentration of 100 ng peptides/mL and with 100 ng/mL LPS, respectively, for 24 h. In inhibitor experiments, 100 µM Tak 242 TLR4 inhibitor (resatorvid, EMD Millipore Corp., Burlington, MA, USA) [51] was added before co-culturing with the peptides for 24 h.

### 2.5. Flow Cytometry

Immunophenotypic analysis was performed by staining the cells with a combination of surface marker antibodies (Table S2) and 7AAD (15239004, 5 µL per sample, ThermoFisher) as well as LIVE/DEAD Fixable Violet (ThermoFisher) in 100 µL PBS for 30 min at 4 °C. For intracellular staining, the cells were washed, fixed and permeabilized using the Foxp3 transcription factor staining buffer set (ThermoFisher) according to the manufacturer’s instructions for 20 min at room temperature (RT) in 100 µL fixation buffer and stained with fluorochrome conjugates against specific cytokines (Table S2) in 100 µL permeabilization buffer for 25 min at RT. Cells were dissolved in 100 µL PBS and immediately acquired on a CytoFlex LX flow cytometer and analyzed with the CytExpert software version 2.6. A lymphocyte/MSC and singlet gate was applied; dead cells and cell debris were excluded. At least 100,000 events were acquired and used for further gating.

### 2.6. Quantification of Inflammatory Cytokines and Chemokines

The Bio-Plex Pro customer-made human cytokine 18-plex bead array (Bio-Rad, Vienna, Austria) was used to quantify a specific set of interleukins (Table S3). The kit includes lyophilized standards to generate standard curves for each analyte with the values measured in the standard dilutions in Table S3 (detection limit of each analyte in S8). Supernatant samples were analyzed undiluted in duplicate according to the manufacturer’s instructions on a Bio-Plex 200 reader (Bio-Rad). Concentrations (pg/mL) of the analytes in the samples were calculated based on the standard curve.

### 2.7. Confocal Microscopy

Confocal microscopy of MSCs treated with SARS-CoV-2 peptides was performed with an Apochromat 63× objective oil on a confocal microscope (TCS SP8, Leica Microsystem GmbH, Wetzlar, Germany) using the LASX-software version 1.4.6. MSCs were cultivated in Nunc Lab-Tek II chamber slides (Nunc, ThermoFisher), fixed and permeabilized using the Foxp3 transcription factor staining buffer set (eBioscience, ThermoFisher) and stained with Alexa Fluor AF 488 phalloidin (1:200, ThermoFisher) for filamentous (f)-actin, MitoTracker Red CMXRos (1:10,000, ThermoFisher) for mitochondria and DAPI (1:1000, Sigma-Aldrich, St. Louis, MO, USA) to stain nuclei. Cells were mounted with Fluoromount-G (ThermoFisher). Images (2048 × 2048 pixels) were analyzed in ImageJ Fiji 1.54p.

### 2.8. Protein Extraction and Western Blot

Cells were resuspended in cold RIPA buffer (ThermoFisher) containing a protease inhibitor cocktail (1:1000, ThermoFisher). Samples were incubated for 30 min on ice and centrifugated at 14,000× *g* for 10 min at 4 °C. Supernatants were collected and protein concentration was determined using a 2100 Bioanalyzer (Agilent, Santa Clara, CA, USA). A total of 6 µg of protein was loaded per sample, run on 4–20% Novex Tris-Glycine gels (Invitrogen) and blotted to a nitrocellulose membrane (ThermoFisher). A total of 2% milk powder (Bio-Rad) in PBS/0.1% Tween-20 (Sigma-Aldrich) was used for blocking and primary antibody dilutions were used in PBS/0.1% Tween-20 overnight agitating at 4 °C. Phospho-p65 (Ser536, 1:1000 dilution, polyclonal, anti-rabbit, 3031S Cell Signaling, Danvers, MA, USA) [52] and GAPDH (1:1000 dilution, polyclonal, anti-rabbit, ab37168, Abcam, Cambridge, UK) [53] were used. Secondary antibody solutions were prepared in PBS/0.1% Tween-20 for horseradish peroxidase (HPR)-conjugated anti-rabbit (1:5000 dilution, 1706515, human IgG adsorbed, Bio-Rad). The detection was performed on a ChemiDoc XRS (Bio-Rad) using Clarity ECL Western Blotting Substrate (Bio-Rad). The marker image was acquired under transmission white light after chemiluminescence detection. The overlay image of chemiluminescence and marker image as well as data analysis was performed in ImageJ. Phospho-p65 expressed is displayed as relative expression to GAPDH.

### 2.9. Statistics

Statistical analyses were performed using GraphPad Prism 7.02. Data were analyzed for normality by Shapiro–Wilk and subsequently statistical differences were assessed by Welch’s test in parametric and the two-tailed Mann–Whitney U-test in non-parametric datasets with small sample sizes. Sample sizes and statistical test used are indicated in the figure legends. *p*-values < 0.05 were considered as statistically significant (*). Data was visualized as box whisker plots, min–max, showing all values, and as a bar graph ± SEM.

## 3. Results

### 3.1. MSCs Exhibited an Altered Immunomodulatory Response When Cocultured with SARS-CoV-2 Peptide-Activated T-Cells

SARS-CoV-2 peptide activated T-cells (T_COVID_) were cocultured with amnion-derived MSCs (Figure S1). The term “COVID” refers to cells stimulated with SARS-CoV-2 peptides. The activation of the T-cells was confirmed by CD69 expression (Figure S2a) and the viability of unstimulated T-cells (T_unstim_) in MSCBM medium compared to RPMI medium was assessed (Figure S2b). All healthy blood donors had received at least one COVID-19 vaccination at the time of the donation. The frequency of SARS-CoV-2 antigen-specific T-cells before vaccination or infection is quite low ranging from 0.1% to 2% [42,54]. After stimulation with the SARS-CoV-2 peptides, we observed a 2.5-fold increase in CD69 on CD3+ T-cells (T_unstim_ mean 3.9 ± SEM 2.19; T_COVID_ mean 9.7 ± SEM 0.79; Figure S2a). In response to T_COVID_, MSCs showed a trend to elevated levels of IL-6 (Figure S3a, mean 23.337 ± SEM 24.725, *n* = 3), IL-1β (Figure S3b, mean 27.877 ± SEM 25.697, n = 3) and tumor necrosis factor (TNF)-α (Figure S3c, mean 5.365 ± SEM 4.581, *n* = 3) compared to coculture with T_unstim_ (IL-6 mean 5.15 ± SEM 4.917, IL-1β mean 7.4 ± SEM 6.521, TNF-α mean 2.263 ± SEM 0.055) (Figure S3a–c). Expression of anti-inflammatory TGF-β (Figure S3d, coculture T_unstim_ mean 0.35 ± SEM 0.485, coculture T_COVID_ mean 0.233 ± SEM 0.127, *n* = 3) and secretion of IL-10 (Figure S3e, coculture T_unstim_ mean 0.4033 ± SEM 0.2517, coculture T_COVID_ mean 0.38 ± SEM 0.3851, *n* = 3) were not altered. Expression of stromal cell-derived factor 1 (SDF-α) is increased in coculture with T-cells comparably between T_unstim_ and T_COVID_ coculture (Figure S3f, coculture T_unstim_ mean 73.11 ± SEM 36.65, coculture T_COVID_ mean 77.4 ± SEM 3.775, *n* = 3). Vascular endothelial growth factor (VEGF) (Figure S3g, coculture T_unstim_ mean 21.39 ± SEM 1.022, coculture T_COVID_ mean 15.91 ± SEM 2.154, *n* = 3) and RANTES (regulated on activation, normal T-cell expressed, and secreted) (Figure S3h, coculture T_unstim_ mean 948.9 ± SEM 696.8, coculture T_COVID_ mean 835.3 ± SEM 192.3, *n* = 3) showed a decrease in coculture with T_COVID_ of 30% and 20%, respectively, while eotaxin was increased in coculture with T-cells but comparable between T_unstim_ and T_COVID_ coculture (Figure S3i, coculture T_unstim_ mean 0.4 ± SEM 0.235, coculture T_COVID_ mean 0.43 ± SEM 0.141, *n* = 3).

In contrast, MSCs cocultured with T-cells activated with the superantigen staphylococcal enterotoxin SEB (T_SEB_) and anti-CD3/CD28 antibodies (T_TCR_) showed slightly increased levels of IL-10, IL-1β and VEGF (Figure S4a–c), with a more pronounced effect observed under TCR stimulation. The chemokine IL-8 is elevated in MSCs in coculture with T_COVID_ and T_TCR_ (Figure S5a,b).

In addition, we analyzed changes in the CD4^+^ FOXP3^+^ population of T_COVID_ in presence of MSCs (Figure S6a). When cocultured with T_COVID_, no significant increase in Treg frequency was observed (Figure S6a, coculture T_unstim_ mean 8.48 ± SEM 4.048, coculture T_COVID_ mean 10.36 ± SEM 5.327). Moreover, Tregs in MSC-T_COVID_ coculture showed a modest reduction in ectonucleotidases CD39 of 30% (Figure S6b, coculture T_unstim_ mean 57.983 ± SEM 28.927, coculture T_COVID_ mean 40.988 ± SEM 19.667), while the expression of intracellular CD73 remained unaltered (Figure S6b, coculture T_unstim_ mean 20.258 ± SEM 8.356, coculture T_COVID_ mean 19.268 ± SEM 7.966). As control, Tregs were also determined in MSC-T_TCR_ and MSC-T_SEB_ cocultures (Figure S6c). In MSC-T_SEB_ coculture, a modest increase in Tregs could be observed compared to coculture with T_unstim_ (Figure S6c, coculture T_unstim_ mean 8.48 ± SEM 4.048, coculture T_SEB_ mean 10.21 ± SEM 1.458), while in presence of T_TCR_, MSCs effectively promoted the expansion of the CD4^+^ FOXP3^+^ Treg subset (Figure S6c, coculture T_unstim_ mean 8.48 ± SEM 4.048, coculture T_TCR_ mean 28.10 ± SEM 13.44).

### 3.2. In Response to SARS-CoV-2 Peptides MSCs Express Higher Levels of TLR4

**Figure 1:**
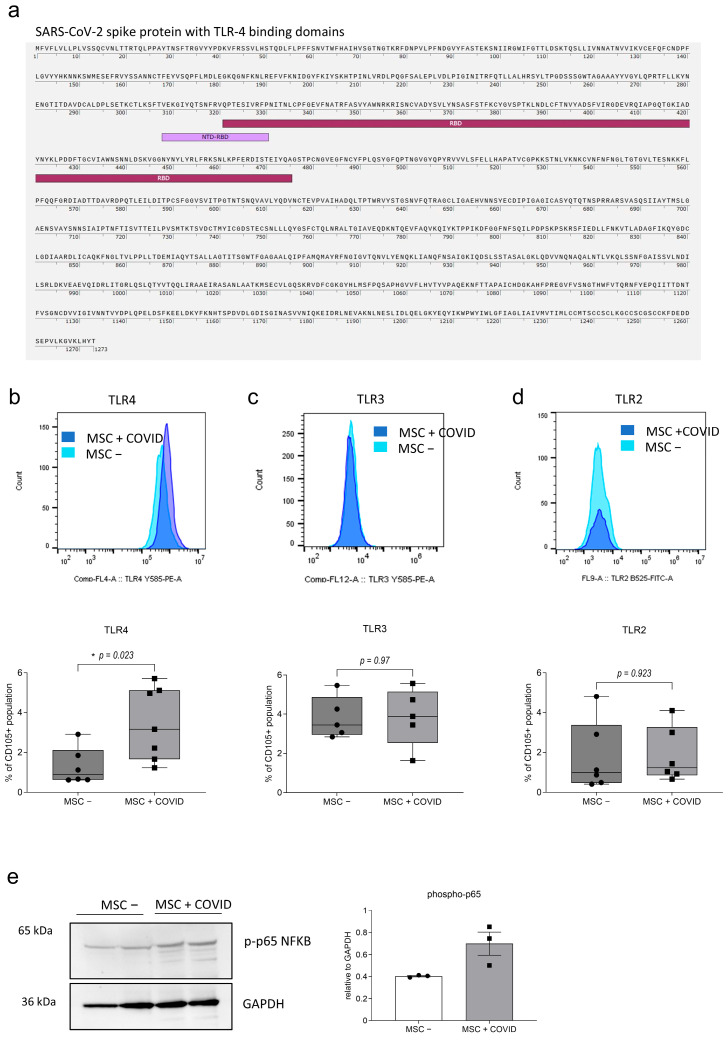
(**a**) Protein sequence of SARS-CoV-2 spike protein (Prot S, NCBI QIH45093.1) highlighting the N-terminal domain (NTD)/receptor binding domain (RBD) (violet) and the RBD core domain (purple). (**b**) TLR4 (*n* = 7), (**c**) TLR3 (*n* = 5) and (**d**) TLR2 (*n* = 6) expression was assessed on MSCs treated with SARS-CoV-2 peptide (MSC + COVID) and unstimulated MSCs (MSC −) by flow cytometry. Data shown as proportion of CD105^+^ living MSCs, box whisker min–max values, Mann–Whitney. Gating of MSCs forward-side scatter (size)-singlets-living-CD105^+^. Representative histograms of each TLR above, MSC − (light blue) and MSC + COVID (dark blue). (**e**) Western blot of two replicates of MSC − and MSC + COVID as well as an analysis of phospho-p65 NFĸB expression relative to GAPDH expression in MSC − and MSC + COVID (*n* = 3). The blot was acquired on a Chemidoc MP Imaging system with an exposure time of 40 s. The blot was processed in ImageJ 1.54p by inverting the image, adjusting the contrast and cropping the specific bands for p-p65 (65 kDa, 1:1000, anti-rabbit) and GAPDH (36 kDa, 1:1000, anti-rabbit). Both p-p65 and GAPDH were analyzed on the same blot using identical processing settings. Values are given as mean ± SEM relative to GAPDH expression. Band sizes indicated in the blot. * *p* < 0.05.

Sequence alignment analysis of the Peptivator Prot S peptides against the human TLR4/MD-2 (myeloid differentiation factor 2) complex (NCBI AAF05316.1 and BAA78717.1) revealed multiple regions of sequence similarity (Table S4). A total of 16 peptide alignments showed 40% sequence identity (six residues out of 15) with the target sequence, while two had sequence identities of 46.6% (seven residues out of 15). Regions within the SARS-CoV-2 spike protein sequence (1273 amino acids) that have been proposed to participate in TLR4 binding are shown in Figure 1a. These include an overlapping region between the N-terminal domain (NTD) and the receptor-binding domain (RBD) highlighted in violet (aa 308–330) as well as the central region of the RBD in purple (aa 320–475). Additionally, 16 peptides from the Peptivator Prot S pool (Table S1) map to these regions.

Stimulated MSCs with SARS-CoV-2-derived peptides (MSC + COVID) were examined for their surface expression of TLR2, TLR3 and TLR4 by flow cytometry (Figure 1b–d, Table S2). TLR4 expression was significantly upregulated in MSC + COVID, showing nearly a two-fold increase compared to untreated MSCs (Figure 1b, MSC − mean 1.3 ± SEM 0.925, *n* = 6; MSC + COVID mean 3.44 ± SEM 1.823, *n* = 7, * *p* 0.023, Mann–Whitney), while TLR3 and TLR2 levels remained unaltered (Figure 1c,d). Moreover, we observed a slight increase in phosphorylated NF-κB p65, a key transcription factor downstream of TLR4, by Western blotting (Figure 1e, original blot is presented in Figure S7). Levels of pp65 NF-κB are displayed relative to GAPDH expression (*n* = 3, MSC − mean 0.403 ± SEM 0.01, MSC + COVID mean 0.699 ± SEM 0.181).

**Figure 2:**
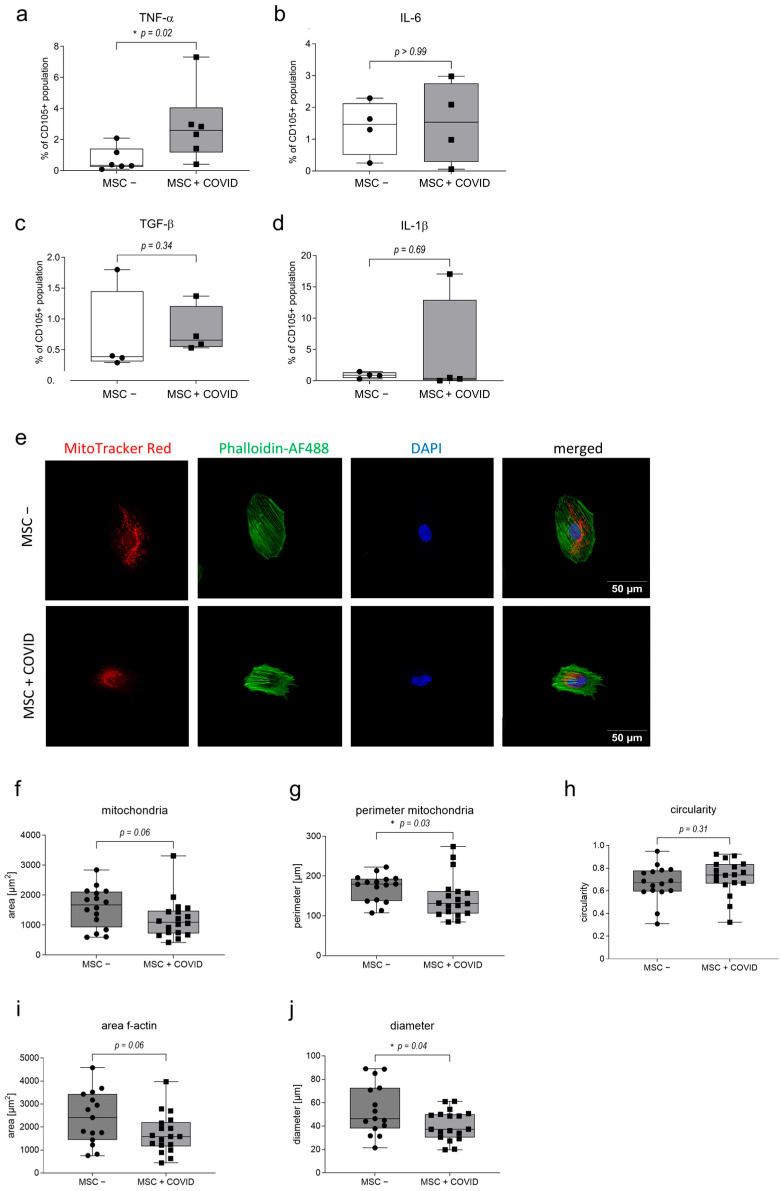
Flow cytometric analysis of intracellular cytokines (**a**) TNF-α (*n* = 6), (**b**) IL-6 (*n* = 4), (**c**) TGF-β (*n* = 4) and (**d**) IL-1β (*n* = 4) in untreated MSCs (MSC −) and MSCs treated with SARS-CoV-2 peptides (MSC + COVID). MSCs were gated by FSC-SSC-singlets-living-CD105^+^. Cytokine expression is displayed as proportion of the living CD105^+^ population. Box whisker min–max values, Mann–Whitney. (**e**) Representative image of MSC − and MSC + COVID stained with MitoTracker Red CMXRos for mitochondria (red), Alexa Fluor AF488 phalloidin for filamentous actin (green), and counterstained with DAPI (nuclei, blue) and analyzed by confocal microscopy (2048 × 2048). The bar indicates 50 μm. (**f**) The area (*n* = 18, Mann–Whitney), (**g**) perimeter (*n* = 18, Mann–Whitney) and (**h**) circularity (*n* = 18, Welch’s test) of mitochondria in MSC − and MSC + COVID were calculated in Image J Fiji. (**i**) The area of filamentous actin (*n* = 18, Mann–Whitney) and (**j**) diameter in µm of each cell (*n* = 18, Mann–Whitney) were measured in Image J Fiji. Box whisker min–max values. * *p* < 0.05.

MSC + COVID also showed a significantly elevated expression of TNF-α (Figure 2a, * *p* = 0.02, Man-Whitney, *n* = 6), while IL-6 was not altered (Figure 2b). TGF-β and IL-1β levels were also comparable between MSC − and MSC + COVID (Figure 2c,d).

In addition, we assessed changes in mitochondra in MSC + COVID cells. There, mitochondria showed a condensed morphology and were redistributed closer to the nucleus (Figure 2e). Based on confocal images of MSC + COVID stained for mitochondria (MitoTracker Red, red), filamentous actin (f-actin, Phalloidin-AF488, green) and the nucleus (DAPI, blue), we observed a decrease in mitochondrial area (Figure 2f, *p* = 0.06, *n* = 16/18, Mann–Whitney) and perimeter (Figure 2g, * *p* = 0.03, *n* = 16/18, Mann–Whitney) in peptide-treated MSCs and a slight increase in circularity (Figure 2h, *p* = 0.31, *n* = 16/18, Welch’s test). Based on f-actin staining, cell area (Figure 2i, *p* = 0.06, *n* = 16/18, Welch’s test) and diameter (Figure 2j, * *p* = 0.04, *n* = 16/18, Welch’s test) were also decreased in MSC + COVID.

### 3.3. MSCs Show an Impaired Immunosuppressive Function on T-Cells in Response to SARS-CoV-2 Peptides

**Figure 3:**
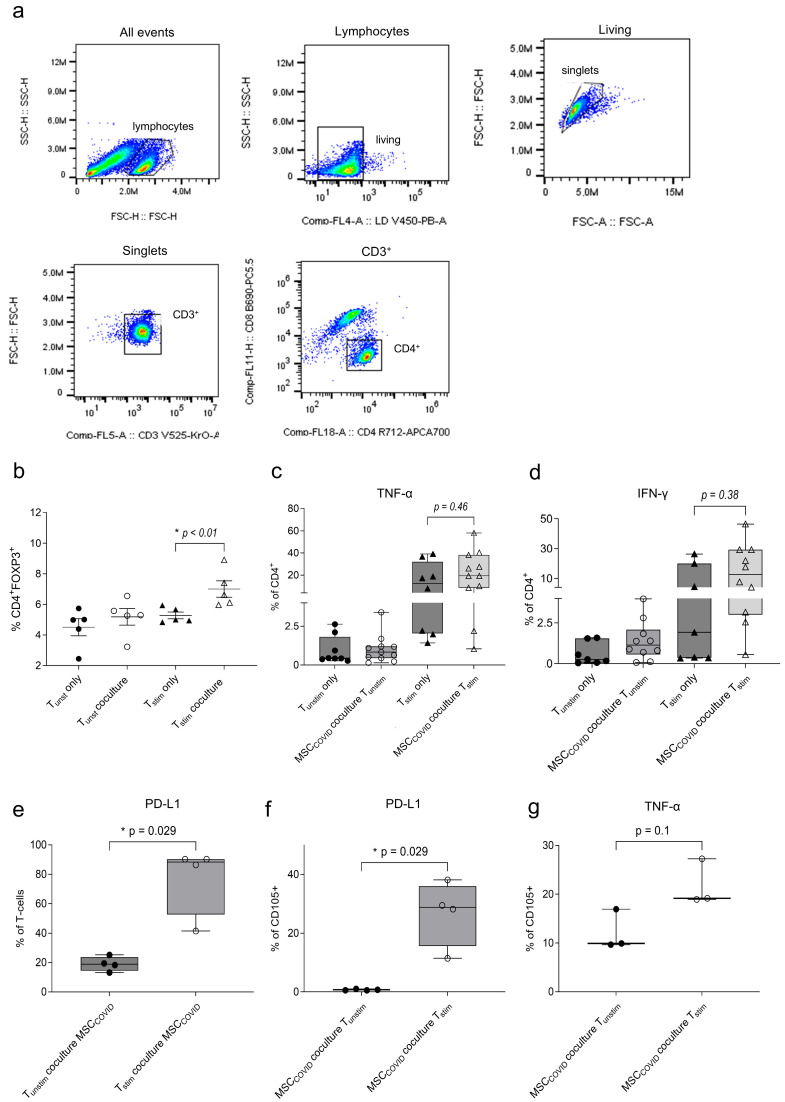
(**a**) Gating strategy of CD4^+^FOXP3^+^ Tregs is displayed. (**b**) The frequency of CD4^+^FOXP3^+^ Tregs was assessed by flow cytometry in CD3^+^ T-cells in coculture with peptide-pretreated MSCs (MSC_COVID_) and as unstimulated (T_unstim_-only) and stimulated (T_stim_-only) single culture (*n* = 5). T-cells were pre-stimulated with TCR stimulant for 24 h before coculture. Flow cytometric analysis of intracellular (**c**) TNF-α (*n* = 11, Mann–Whitney), (**d**) IFN-γ (*n* = 10, Mann–Whitney) in CD4^+^ T-cells (gating as in a) in coculture with peptide-pretreated MSCs (MSC_COVID_). PD-L1 on the surface of (**e**) T-cells (*n* = 4, Mann–Whitney) and (**f**) CD105^+^ MSC_COVID_ (*n* = 4, Mann–Whitney) and (**g**) intracellular level of TNF-α in CD105^+^ MSC_COVID_ (*n* = 3, Mann–Whitney) in coculture with T_unstim_ and T_stim_ were measured by flow cytometry. Box whisker min–max values. MSC gating forward-side scatter-singlets-living-CD105^+^. * *p* < 0.05.

In coculture with MSC_COVID_, the CD4^+^ FOXP3^+^ population in CD4^+^ T-cells (Figure 3a) was significantly increased in T_stim_ in coculture with peptide-primed MSCs compared to T_stim_-only cultures (Figure 3b, * *p* < 0.01, *n* = 5, Mann–Whitney). TNF-α (Figure 3c; T_stim_ mean 15.676 ± SEM 15.268, *n* = 8; MSC_COVID_ coculture T_stim_ mean 22.834 ± SEM 17.630, *n* = 11) and IFN-γ (Figure 3d; T_stim_ mean 7.7 ± SEM 10.872, n = 7; MSC_COVID_ coculture T_stim_ mean 16.303 ± SEM 15.366, *n* = 10) expression in the CD4^+^ T-cell population remained comparable to T_stim_ with a tendency towards an elevated cytokine expression. Significantly increased programmed death (PD)-ligand(L)1 levels on the cocultured T_stim_ (Figure 3e, * *p* = 0.029, *n* = 4, Mann–Whitney) as well as on MSC_COVID_ in coculture with T_stim_ (Figure 3f, * *p* = 0.029, *n* = 4, Mann–Whitney) could be observed. TNF-α expression was further upregulated around 2-fold in MSC_COVID_ upon coculture with T_stim_ (Figure 3g; T_unstim_ coculture mean 12.17 ± SEM 4.133; T_stim_ coculture mean 21.787 ± SEM 4.741, *n* = 3).

MHC class II (HLA-DR, Figure S8a, MSC − mean 3.826 ± SEM 2.262, MSC_COVID_ mean 4.984 ± SEM 3.025, *n* = 5) and MHC class I (HLA-A, -B, -C, Figure S8b, MSC − mean 98.69 ± SEM 0.176, MSC_COVID_ mean 98.61 ± SEM 0.423, *n* = 3) antigen remained unaltered in MSC_COVID_.

In coculture with SEB- and TCR-activated Th-cells (CD3^+^CD4^+^, CD3^+^CD5^+^ T-cells), MSCs suppress the expression of the pro-inflammatory cytokines TNF-α and IFN-γ (Figure S9a–d). In presence of MSCs, T_TCR_ showed a reduction in TNF-α of 35% (Figure S9a, T_TCR_ mean 20.69 ± SEM 17.14, T_TCR_ coculture mean 13.08 ± SEM 10.13, *n* = 6) and a 30%–decrease in IFN-γ (Figure S9b, T_TCR_ mean 10.3 ± SEM 6.814, T_TCR_ coculture mean 7.81 ± SEM 3.755, *n* = 7). Notably, T_SEB_ showed a significant increase in TNF-α in presence of MSCs compared to T_SEB_-only (Figure S9c, *p* = 0.02, *n* = 5, Mann–Whitney), but an unaltered level of IFN-γ (Figure S9d).

### 3.4. The Impaired Immunosuppressive Function of MSCs in Response to SARS-CoV-2 Peptides Is Associated with TLR4-Signaling

**Figure 4:**
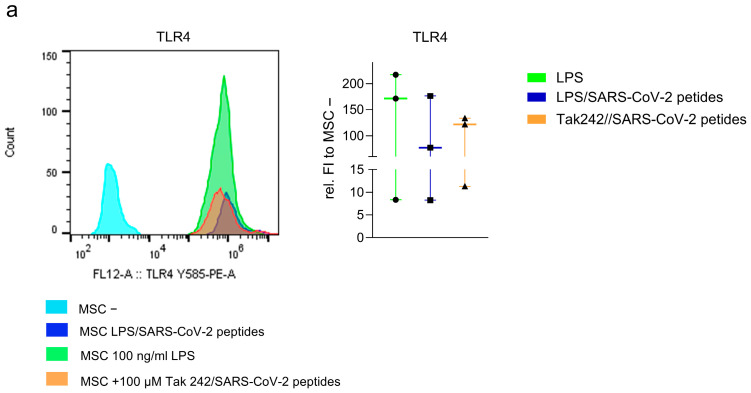

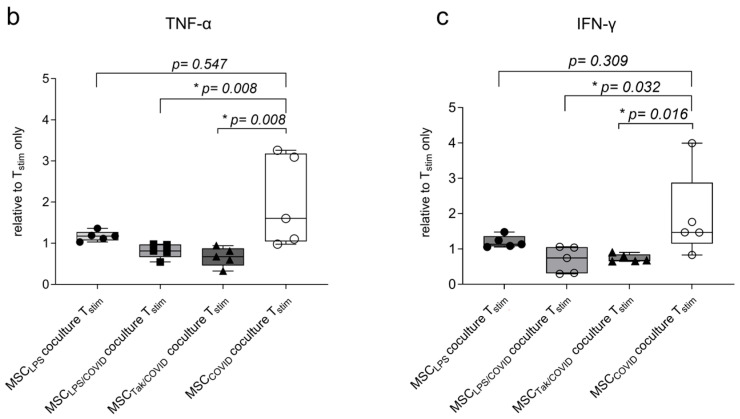
(**a**) Representative flow cytometry histogram of TLR4 and relative fluorescent intensity of TLR4 in treated MSCS relative to untreated MSC (*n* = 3). Untreated MSCs (MSC −, light blue), MSCs treated with 100 ng/mL LPS and 100 ng/mL SARS-CoV-2 peptides (dark blue), MSCs treated with 100 ng/mL LPS alone (green), and MSCs treated with 100 µM TAK-242 inhibitor and 100 ng/mL SARS-CoV-2 peptides (orange) for 24 h. MSC gating: size gate-singlets-living-CD105^+^. (**b**) Flow cytometric analysis of intracellular (**b**) TNF-α (*n* = 5, Mann–Whitney), and (**c**) IFN-γ (*n* = 5, Mann–Whitney) in CD4^+^ T-cells (gating lymphocytes-singlets-living-CD3^+^CD4^+^CD8^−^) in coculture with MSC pre-treated with 100 ng/mL LPS (MSC_LPS_), with 100 ng/mL LPS and 100 ng/mL SARS-CoV-2 peptides (MSC_LPS/COVID_) and with 100 µM Tak 242 TLR4 inhibitor and 100 ng/mL SARS-CoV-2 peptides (MSC_Tak/COVID_) for 24 h. T-cells were pre-stimulated with TCR for 24 h before coculture. Values are presented as proportion of CD4^+^ T-cell population relative to T_stim_. Five representative experiments from Figure 3c,d were integrated and displayed as proportion in CD4^+^ T-cells relative to T_stim_. Box whisker min–max values. * *p* < 0.05.

To assess the involvement of TLR4 signaling in the impact of SARS-CoV-2 peptides on MSC immunomodulation, we analyzed TLR4 expression in MSCs following 100 ng/mL LPS stimulation, a combination of LPS and SARS-CoV-2 peptides as well as treatment with the 100 µM TLR4 inhibitor Tak 242 in combination with SARS-CoV-2 peptides for 24 h. Tak 242 inhibitor (resatorvid) downregulates expression of TLR4 downstream signaling molecules MyD88 and TRIF [54]. Representative flow cytometry histograms (Figure 4a) from three independent experiments show low basal TLR4 expression in untreated MSC −, whereas stimulation with LPS resulted in a strong upregulation of TLR4 on CD105^+^ MSCs. An increased TLR4 expression was also observed in MSCs treated with LPS in combination with SARS-CoV-2 peptides. Treatment with TAK 242 in the presence of SARS-CoV-2 peptides reduced TLR4 expression.

Next, we evaluated the functional consequences of MSC pretreatment on CD4^+^ T-cell cytokine production in coculture. MSCs were pretreated for 24 h with LPS (MSC_LPS_), LPS and SARS-CoV-2 peptides (MSC_LPS/COVID_) and TAK-242 and SARS-CoV-2 peptides (MSC_TAK/COVID),_ respectively, and subsequently cocultured with TCR-stimulated T-cells (T_Stim_). Flow cytometric analysis of the intracellular cytokines TNF-α (Figure 4b) and IFN-γ (Figure 4c) was performed in cocultured CD4^+^ T-cells compared to T_stim_-only. The figure also includes data from Figure 3, showing the cytokine expression in MSC_COVID_-T_stim_ coculture relative to T_stim_ for comparison. MSC_LPS_ showed similar effects on cytokine expression than MSC_COVID_. In contrast, coculture with MSC_TAK/COVID_ reduced TNF-α to 67% and IFN-γ expression to 73% relative to T_stim_ and differed significantly from MSC_COVID_ cocultures (Mann–Whitney, *n* = 5, TNF-α *p* = 0.008, IFN-γ *p* = 0.016). MSC_LPS/COVID_ exhibited an immunosuppressive effect on T-cells comparable to that of MSC_Tak/COVID_ reducing TNF-α levels to 80% and IFN-γ to 70% in coculture relative to T_stim_-only culture, and significantly lower than in MSC_COVID_ coculture (Figure 4b,c, *n* = 5, Mann–Whitney, TNF-α *p* = 0.008, IFN-γ *p* = 0.032).

## 4. Discussion

MSCs have gained substantial attention for their ability to alleviate inflammatory conditions due to their well-established immunosuppressive properties [27]. Therefore, MSCs have potential in mitigating inflammatory responses and supporting tissue repair in COVID-19 infections.

In line with this, our data indicates that MSCs respond to SARS-CoV-2 peptides by adopting a pro-inflammatory phenotype. This reprogramming may be initiated through the binding of these peptides to the TLR4 surface receptor [13], with direct consequences for their immunoregulatory capacity. The observed morphological changes, like mitochondrial condensation with perinuclear clustering and increased cytoskeletal density, in MSCs treated with SARS-CoV-2 peptides are consistent with cellular stress and inflammatory activation. These alterations likely reflect metabolic reprogramming and increased cytoskeletal tension, both of which have been linked to altered secretory behavior and reduced immunomodulatory function [55,56].

Under physiological conditions, MSCs can suppress effector T-cell responses, inhibit cytokine production, and promote Treg expansion [57,58]. MSCs pretreated with SARS-CoV-2 peptides exhibited impaired suppression of T-cell cytokine expression and failed to significantly downregulate TNF-α and IFN-γ in CD4^+^ T-cells. Moreover, exposure to SARS-CoV-2 peptides resulted in increased TLR4 expression and activation of NF-κB signaling in MSCs, accompanied by upregulation of TNF-α, whereas anti-inflammatory cytokines such as TGF-β and IL-10 were not induced. These findings are consistent with previous reports demonstrating that TLR4 stimulation polarizes MSCs toward a pro-inflammatory phenotype characterized by reduced immunosuppressive function and altered secretory profiles [11,59].

Although a direct interaction between SARS-CoV-2 peptides and TLR4 cannot be conclusively demonstrated in this study, our findings suggest the potential involvement of TLR4 signaling. This is supported by in silico and experimental studies reporting sequence similarities between SARS-CoV-2 spike peptides and known TLR4-interacting motifs [41,60,61], as well as the identification of spike protein domains implicated in TLR4 binding. Importantly, inhibition of TLR4 prevented the peptide-induced impairment of MSC-mediated cytokine suppression in T-cells, supporting a TLR4-dependent mechanism. Similarly, LPS-stimulated MSCs showed increased TLR4 expression and a reduced capacity to suppress T-cell cytokine production, comparable to peptide-treated MSCs. Interestingly, concurrent exposure of MSCs to LPS and SARS-CoV-2 peptides resulted in a physiological repression of pro-inflammatory cytokine production in T-cells, suggesting a potential reciprocal inhibitory interaction between LPS- and peptide-mediated signaling pathways.

To further investigate MSC–T-cell interactions under these conditions, we analyzed the expression of immune checkpoint molecules. Peptide-treated MSCs exhibited significantly elevated PD-L1 expression in coculture with stimulated T-cells, which was mirrored by increased PD-L1 levels on T-cells cocultured with these MSCs. Additionally, TNF-α expression was significantly upregulated in MSCs during coculture with activated T-cells. Consistent with the established role of PD-L1 in promoting Treg differentiation [62], an increased Treg frequency was observed in T-cells cocultured with peptide-pretreated MSCs. PD-L1 can bind both PD-1 and CD80, with distinct functional consequences. The PD-L1/PD-1 interaction delivers inhibitory signals that suppress T-cell activation and cytokine production, whereas PD-L1 binding to CD80 can inhibit PD-1/PD-L1 signaling while maintaining CD80-CD28 interactions that promote T-cell activation [63,64].

T-cells stimulated with SARS-CoV-2 peptides showed only a moderate increase in CD4^+^FOXP3^+^ Tregs in the presence of MSCs. Moreover, these cells lacked upregulation of CD39 and CD73, key mediators of adenosine-dependent Treg suppressive function [65], indicating impaired anti-inflammatory capacity. Although purified T-cells were used, a small fraction of contaminating APCs likely remained, as T-cell purity typically ranges from 90% to 97%. Even low numbers of APCs are sufficient to support antigen presentation and activation of antigen-specific T-cells [66]. SARS-CoV-2 peptides consist of overlapping 15-mer peptides designed for direct loading onto MHC class I and II molecules without prior processing, thereby enabling antigen presentation by residual APCs. Effective T-cell stimulation was confirmed by assessing CD69 expression following peptide exposure relative to untreated controls. MSCs do not constitutively express MHC class II molecules, and exposure to SARS-CoV-2 peptides did not induce MHC class II upregulation in our study. Furthermore, MSC-mediated suppression of T-cell responses has been consistently reported even in HLA-mismatched settings [67,68].

While our in vitro experiments cannot fully recapitulate the complex environment of severe COVID-19, our results suggest that SARS-CoV-2 antigenic stimulation involving TLR4 signaling can modulate the anti-inflammatory properties of MSCs and represent one of several mechanisms contributing to immune dysregulation in COVID-19 patients. A limitation of this study is that the data presented indicate trends rather than definitive statistically significant outcomes, likely reflecting differences in cellular responsiveness to the peptides. Further investigations are required to clarify the mechanisms underlying receptor activation in response to SARS-CoV-2 peptides and to determine whether the observed MSC phenotype is specific to SARS-CoV-2 or may also be induced by other viral antigens.

Overall, our findings support the emerging concept of MSC plasticity, whereby external cues such as TLR ligands or viral antigens can reprogram MSC behavior in ways that either enhance or impair inflammatory responses [58,69]. Given the reported potential of MSCs to modulate cytokine responses and support lung regeneration in COVID-19 [26], our data underscores the importance of understanding how inflammatory cues influence MSC function.

## 5. Conclusions

In summary, our data indicates that MSCs can respond to SARS-CoV-2-derived peptides in a TLR4-associated manner, resulting in altered immunosuppressive activity and changes in T-cell regulation. Given the central role of T-cells in COVID-19 pathogenesis where dysregulated effector responses and impaired regulatory T-cell function contribute to increased inflammation, these findings provide mechanistic insight into how SARS-CoV-2 antigens can influence MSC plasticity and, consequently, shape T-cell responses.

## Figures and Tables

**Table 1:** Donor characteristics.

Group	Blood Donations	Placental Tissue
Number of subjects	16	18
Age range (y), median	26–63, 40.5	not available *
Sex (*n*, %)		
male	8 (50)	
female	8 (50)	18 (100)

* Not covered by the ethical approval.

## Data Availability

The original data presented in the study are openly available in the repositorium DOOR at https://doi.org/10.48341/vrdk-5755 (accessed on 18 March 2025).

## Supplementary Materials

The following supporting information can be downloaded at: https://www.mdpi.com/article/10.3390/cells15070592/s1, Figure S1: Representative histograms of MSC positive and negative surface markers; Figure S2: Flow cytometric analysis of a) CD69 expression in T_COVID_ and b) viability of T-cells in MSCBM medium; Figure S3: Analysis of inflammatory mediators in COVID cocultured cells and supernatants; Figure S4: Analysis of inflammatory mediators in SEB and TCR cocultured supernatants; Figure S5: Flow cytometric staining of intracellular IL-8 in cocultured MSCs; Figure S6: Flow cytometric analysis of regulatory T-cell population in cocultured T_COVID_, T_TCR_ and T_SEB_; Figure S7: Western blot of NF-κB in MSC + COVID; Figure S8: Analysis of HLA type I and type II expression on MSC + COVID; Figure S9: Flow cytometric analysis of intracellular cytokines in cocultured T_TCR_ and T_SEB_; Table S1: List of peptide sequences in the Peptivator SARS-CoV-2 Prot S peptide pool from Miltenyi; Table S2: List of fluorochrome conjugates used in flow cytometric staining; Table S3: Bio-Plex Pro Human Cytokine Screening Panel, 18-Plex, analytes with region and value; Table S4: Alignment of Peptivator Prot S peptide sequences with the TLR4-MD-2 protein sequence by SnapGene.
