# Supplementary material for: Mesenchymal Stromal Cells Respond to SARS-CoV-2 Peptides and Exhibit Altered T-Cell Regulatory Capacity
Source: Cells. 2026 Mar 26;15(7):592. doi: 10.3390/cells15070592 (PMC13072370; doi:10.3390/cells15070592)
Supplement: Supplementary file 1 [file cells-15-00592-s001.zip › Supplementary Material.pdf]

Supplementary Material

Mesenchymal stem cells respond to SARS-CoV-2 peptides and exhibit altered T-cell regulatory capacity

Sabrina Summer[1,*], Hermann M. Wolf[3], Viktoria Weber[2], Michael B. Fischer[1,4]

[1]Center for Experimental Medicine, Department for Biomedical Research, University for Continuing Education Krems, Krems, Austria

[2]Center for Biomedical Technology, Department for Biomedical Research, University for Continuing Education Krems, Krems, Austria

[3] Faculty of Medicine, Sigmund Freud Private University, Vienna, Austria

[4]Department for Blood Group Serology and Transfusion Medicine, Medical University of Vienna, Vienna, Austria

Supplementary Figures

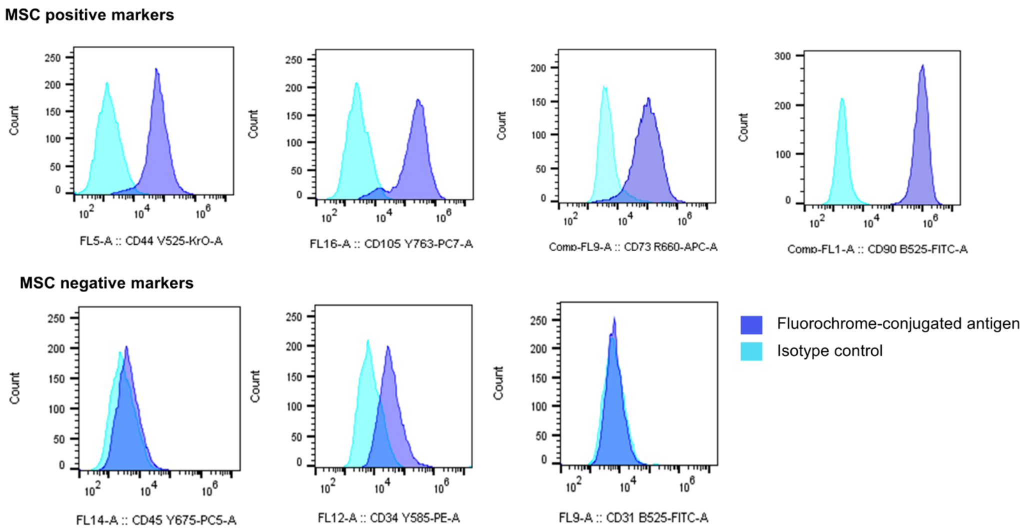

Figure S1. Representative histograms of MSC positive and negative surface markers. Positive surface marker CD44, CD105, CD73 and CD90 as well as negative marker CD45, CD34 and CD31 were measured by flow cytometry (dark blue). The histograms present results of human amnion-derived MSCs in passage 1. The CD marker antigens were tagged with different fluorochromes (x-axis). The histograms of the positive markers were considerably shifted to the right compared to their respective isotype controls (light blue).

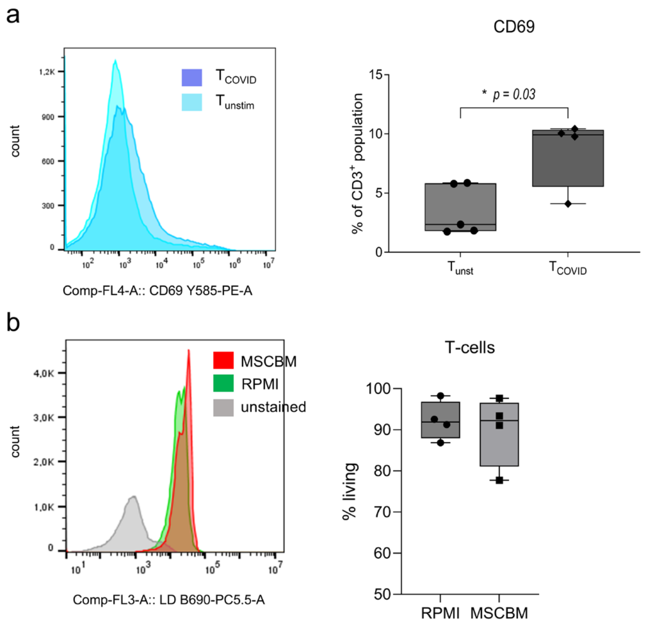

Figure S2. Flow cytometric analysis of a) CD69 expression in T_{COVID} and b) viability of T-cells in MSCBM medium. Histogram of CD69 (left) on T_{unstim} (light blue) and T_{COVID} (dark blue) in gated $CD3^+$ T-cells unstimulated (T_{unst}) and stimulated with SARS-CoV-2 peptides for 24 hours (T_{COVID}). Gating: lymphocytes-singlets-living-$CD3^+$. Percentage of T-cells expressing CD69 in T_{COVID} compared to $T_{unst.}$ Box whisker min–max values (*n*=5/4, Mann–Whitney). b) Viability of T_{unstim} grown for 20 hours in RPMI and MSCBM medium both supplemented with 10% FBS. Histogram of 7AAD in unstained T-cells (gray), stained T-cells grown in RPMI medium (green) and in MSCBM medium (red). Percentage of T-cells expressing 7AAD (%living) in T-cell size gate visualized in box whisker min–max values (*n*=4).

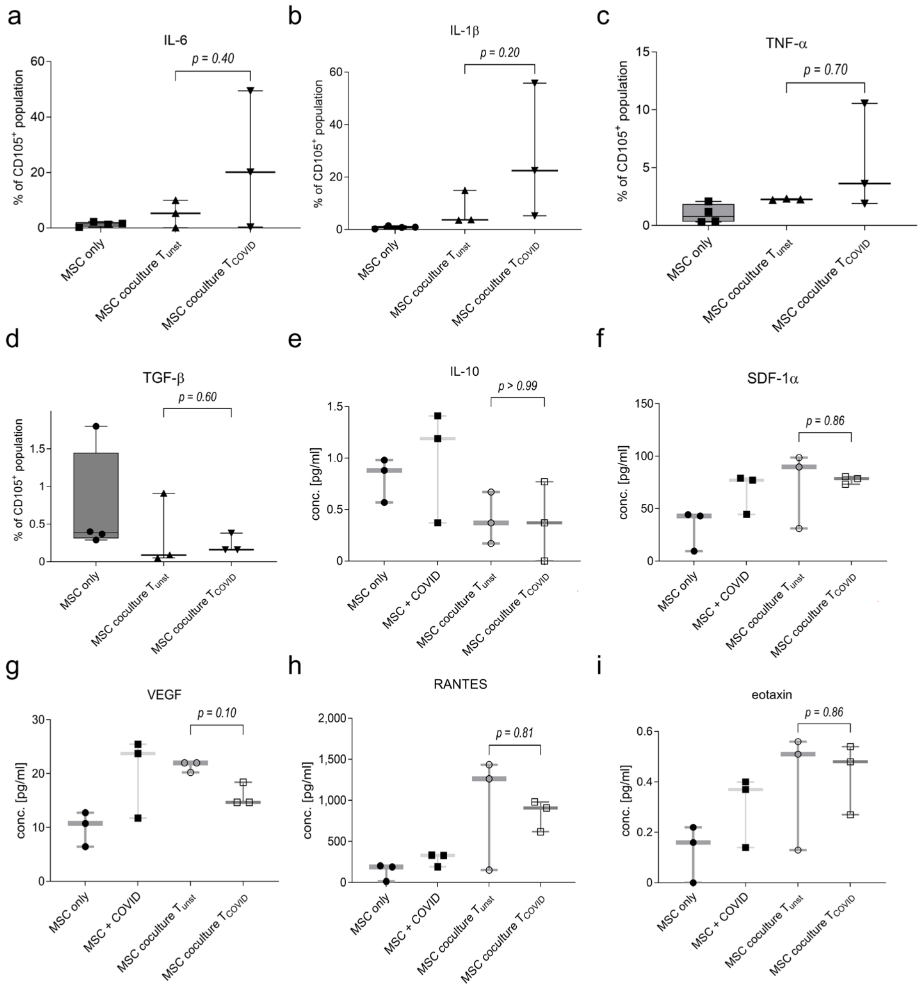

Figure S3. : Analysis of inflammatory mediators in COVID cocultured cells and supernatants. Flow cytometric analysis of intracellular a) IL-6, b) IL-1β, c) TNF-*α* and d) TGF-β in MSCs cocultured with T-cells stimulated with SARS-CoV-2 peptides. Single MSC cultures and MSCs in coculture with unstimulated (T_{unst}) and SARS-CoV-2 peptide-stimulated T-cells (T_{COVID}) were analyzed for intracellular cytokine expression. MSCs were gated by FSC-SSC (size gate)-singlets-living-$CD105^+$. Cytokine expression is displayed as proportion of the living $CD105^+$ population (n=3, Box whisker min–max values, Mann–Whitney). Analysis of bead-based multiplex assay of inflammatory mediators in coculture supernatants. Concentrations of (e) IL-10, (f) SDF1-*α*, (g) VEGF, (h) RANTES and (i) eotaxin were determined in the supernatant of single MSC cultures and coculture with T_{COVID} using a bead-based multiplex assay (Bio-Plex Pro human cytokine 18-plex bead array from Bio-Rad). Box whisker min–max values (n=3, Mann–Whitney). All samples were analyzed undiluted in duplicate on a Bio-Plex 200 reader (Bio-Rad). Concentrations (pg/mL) of the analytes in the samples were calculated based on a standard curve.

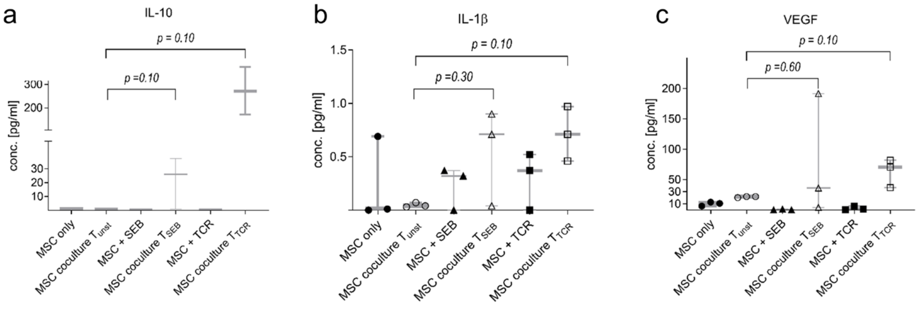

Figure S4. Analysis of inflammatory mediators in SEB and TCR cocultured supernatants. Concentrations of (a) IL-10, (b) IL-1β and (c) VEGF were measured in the supernatant of SEB- and TCR-stimulated single MSC as well as cocultures with T_{SEB} and T_{TCR} using a bead-based multiplex assay (Bio-Plex Pro human cytokine 18-plex bead array from Bio-Rad). Box whisker min−max values (*n*=3, Mann−Whitney). All samples were analyzed undiluted in duplicate on a Bio-Plex 200 reader (Bio-Rad). Concentrations (pg/ml) of the analytes in the samples were calculated based on a standard curve.

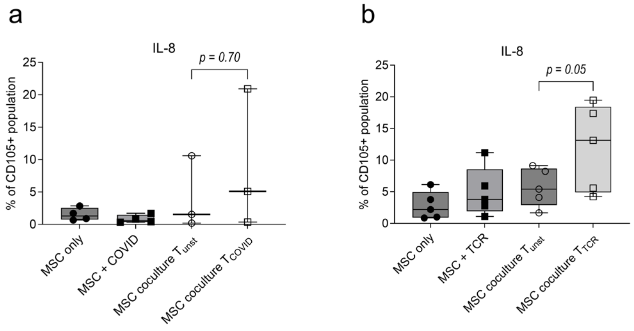

Figure S5. Flow cytometric staining of intracellular IL-8 in cocultured MSCs. Single MSC culture and MSCs cocultured with unstimulated (T_{unst}) and SARS-CoV-2 peptide-stimulated T-cells (T_{COVID}) (a) and TCR-stimulated T-cells (T_{TCR}) (b) for 20 hours were analyzed for IL-8 expression. MSCs were gated following FSC/SSC size-singlets-living-CD105^{+}, and the cytokine expression is displayed as proportion of the living CD105^{+} population (n=4, Mann-Whitney). Box whisker min.-max values.

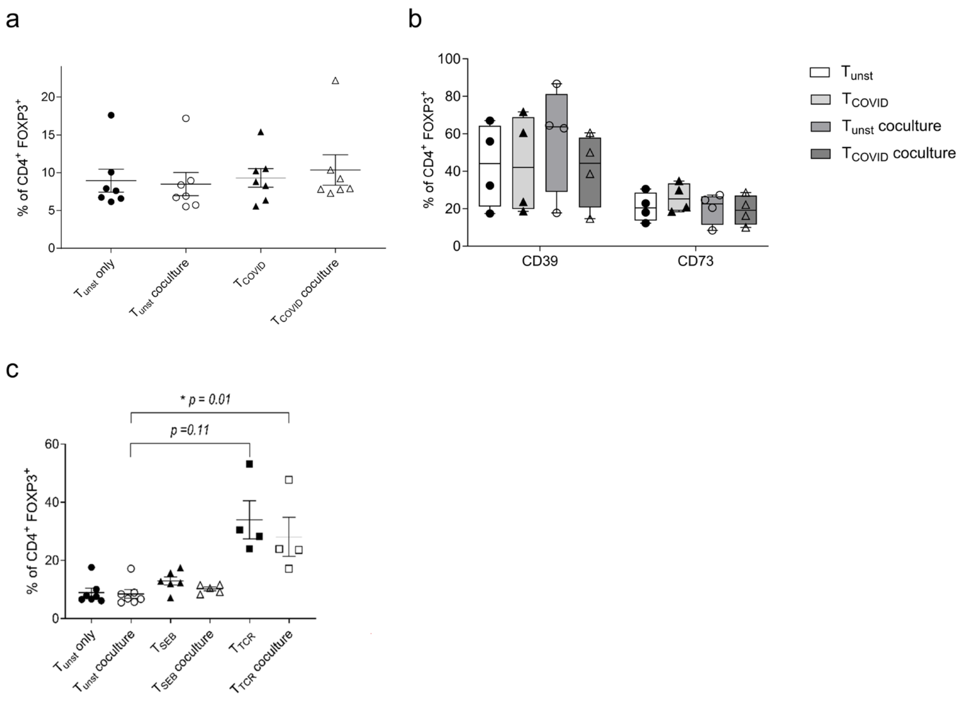

Figure S6. Flow cytometric analysis of regulatory T-cell population in cocultured T_{COVID}, T_{TCR} and T_{SEB}. a) Frequency of regulatory T-cells (Tregs, $CD4^+$ $FOXP3^+$) was assessed by flow cytometry within $CD3^+$ T-cells treated with SARS-CoV-2 peptides for 24 hours before coculturing with MSCs as well as in the unstimulated and stimulated single culture (n = 7). Treg population gating lymphocytes-singlets-living-$CD3^+$-$CD4^+$-$FOXP3^+$. (b) In this Treg population the expression of intracellular CD73 and surface CD39 was determined by flow cytometry and displayed as proportion in the $CD4^+$ $FOXP3^+$ population before and after coculture and as single culture (n = 4). Box whisker min–max values. (c) Frequency of regulatory T-cells (Tregs, $CD4^+FOXP3^+$) was assessed by flow cytometry within $CD3^+$ T-cells treated with SEB and TCR for 24 hours before coculturing with MSCs (n = 4) as well as in unstimulated (n = 7) and stimulated (n = 6) single T-cell cultures. Mann–Whitney.

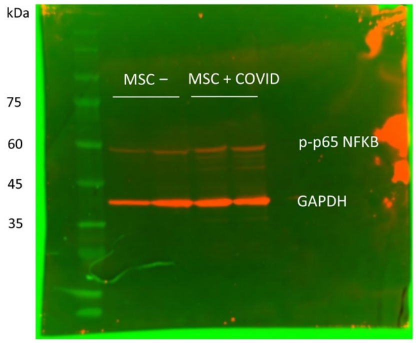

Figure S7. Western blot of NF-κB in MSC +COVID. Western blot analysis of phospho-p65 (p-p65) NF-κB in MSCs treated with SARS-CoV-2 peptides. GAPDH was used as control. Original image (pixel 696x517, 201.1μm x 201.1 μm) acquired on a ChemiDoc MP Imaging system using 40 seconds exposure time. As ladder the pre-stained protein marker 10-180 kDa was used, acquired in transmission white mode. Overlay performed in Image J, chemiluminescence signal in red, marker in green.

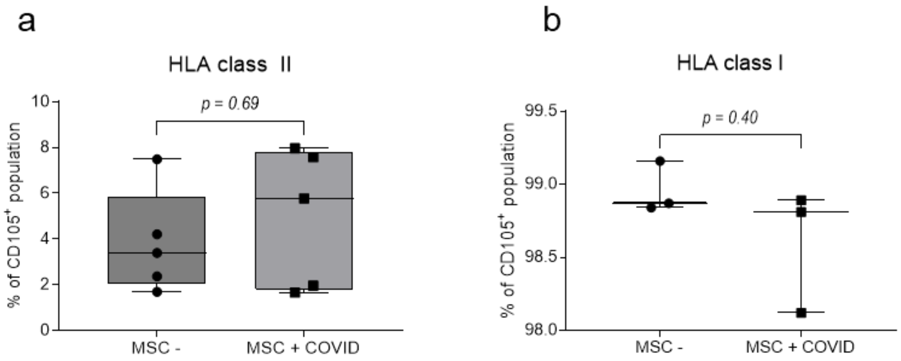

Figure S8. Analysis of HLA type I and type II expression on MSC + COVID. Flow cytometric staining for (a) MHC class II antigen (HLA-DR) and (b) MHC class I antigen (HLA-A,B,C) in untreated MSC − and SARS-CoV-2 peptide-treated MSCs. MSC gating MSC forward-side-scatter size gate-singlets-living-CD105^{+}. Box whisker min.-max. values (n=5/n=3, Mann−Whitney).

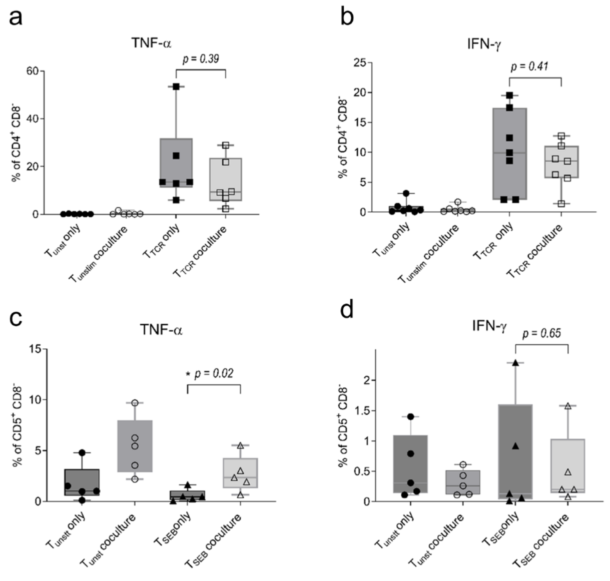

Figure S9. Flow cytometric analysis of intracellular cytokines in cocultured T_{TCR} and T_{SEB}. a,c) TNF-α and b,d) IFN-γ expression levels were assessed in $CD3^{+}CD5^{+}$ $CD8^{-}$ T-cells prestimulated with a,b) TCR reagent and c,d) SEB antigen for 24 hours in single T-cell culture as well as in coculture with MSCs for 20 hours. Gating T-cells lymphocytes-singlets-living-$CD3^{+}$-$CD5^{+}CD8^{-}$. Box whisker min–max. values (n=5, Mann−Whitney).

Supplementary Tables

Supplementary Table S1. List of peptide sequences in the Peptivator SARS-CoV-2 Prot S peptide pool from Miltenyi

Supplementary Table S2. List of fluorochrome conjugates used in flow cytometric staining

Supplementary Table S3. Bio-Plex Pro Human Cytokine Screening Panel, 18-Plex, analytes with region and value.

Analyte	Region	S1	S2	S3	S4	S5	S6	S7	S8
		[pg/ml]							
Eotaxin	(43)	2683	670,8	167,7	41,9	10,5	2,6	0,7	0,2
GM-CSF	(34)	6547	1636,8	409,2	102,3	25,6	6,4	1,6	0,4
IFN-γ	(21)	20392	5098,0	1274,5	318,6	79,7	19,9	5,0	1,2

IL-1b	(39)	7478	1869,5	467,4	116,8	29,2	7,3	1,8	0,5
IL-1a	(63)	51660	12915,0	3228,8	807,2	201,8	50,4	12,6	3,2
IL-4	(52)	3449	862,3	215,6	53,9	13,5	3,4	0,8	0,2
IL-6	(19)	3964	991,0	247,8	61,9	15,5	3,9	1,0	0,2
IL-8	(54)	8348	2087,0	521,8	130,4	32,6	8,2	2,0	0,5
HGF	(62)	248746	62186,5	15546,6	3886,7	971,7	242,9	60,7	15,2
IL-10	(56)	11807	2951,8	737,9	184,5	46,1	11,5	2,9	0,7
IL-17A	(76)	42611	10652,8	2663,2	665,8	166,4	41,6	10,4	2,6
IP-10	(48)	26288	6572,0	1643,0	410,8	102,7	25,7	6,4	1,6
MCP-1	(53)	8948	2237,0	559,3	139,8	35,0	8,7	2,2	0,5
MIG	(14)	42599	10649,8	2662,4	665,6	166,4	41,6	10,4	2,6
SDF-1a	(22)	54844	13711,0	3427,8	856,9	214,2	53,6	13,4	3,3
RANTES	(37)	18436	4609,0	1152,3	288,1	72,0	18,0	4,5	1,1
TNF-a	(36)	103197	25799,3	6449,8	1612,5	403,1	100,8	25,2	6,3
VEGF	(45)	110471	27617,8	6904,4	1726,1	431,5	107,9	27,0	6,7
Dilutions:			*1:4*	*1:4*	*1:4*	*1:4*	*1:4*	*1:4*	*1:4*

Supplementary Table S4. Alignment of Peptivator Prot S peptide sequences with the TLR4-MD-2 protein sequence by SnapGene.
